# The impact of environmental protection tax on the health of middle-aged and older adults: evidence from CHARLS data in China

**DOI:** 10.3389/fpubh.2024.1446248

**Published:** 2024-08-21

**Authors:** Hao Wang, Ruifan Zhou, Haohan Luo, Shanwen Liang, Youzhuan Kong

**Affiliations:** ^1^China School of Banking and Finance, University of International Business and Economics, Beijing, China; ^2^Faculty of Economics, China Sichuan Administration Institute, Chengdu, China; ^3^Business School, Chengdu University of Technology, Chengdu, China; ^4^Cntic-Vpower Business Department, China National Technical Import and Export Corp., Beijing, China; ^5^School of History, Geography and Tourism, Chengdu Normal University, Chengdu, China

**Keywords:** environmental protection tax, ecological environment, public health, policy evaluation, difference-in-differences model

## Abstract

**Introduction:**

With the aging population, the relationship between human health and the ecological environment has gained increasing attention. In China, it is imperative to evaluate the policy effects of the Environmental Protection Tax (EPT) on improving the ecological environment and enhancing the health of middle-aged and older adult people.

**Methods:**

This study, based on data from the China Health and Retirement Longitudinal Study (CHARLS), employs a Difference-in-Differences (DID) model to assess the health effects of the EPT policy.

**Results:**

The findings indicate that the EPT policy significantly improves the health of middle-aged and older adult individuals and reduces the prevalence of chronic diseases. The EPT policy affects the health of middle-aged and older adult through two main mechanisms: emission reduction and psychological effects. These are evidenced by reductions in PM10 particle concentration and sulfur dioxide emissions, improvements in public sleep quality and memory, and significant changes in environmental awareness and concern.

**Discussion:**

Heterogeneity analysis reveals differences across urban and rural areas, age groups, and education levels. Following the implementation of the EPT policy, there are notable improvements in reduction of chronic diseases among rural residents, self-rated health among urban residents, and overall health among the older adult and individuals with a junior high school education or lower. The study’s results confirm the importance of environmental policies in promoting public health, providing a reference for the refinement of the EPT system, and offering insights for environmental pollution control in developing countries.

## 1 Introduction

China is rapidly entering a phase of deep aging, with environmental pollution factors having long-term impacts on the health of middle-aged and older adult individuals, exacerbating health risks such as respiratory and cardiovascular diseases. Ensuring the health and well-being of the middle-aged and older adult has become a significant challenge in China’s new stage of development. Establishing a good ecological environment and safeguarding human health is of utmost urgency. In China’s 13th Five-Year Plan, “Beautiful China,” and “Healthy China” emerged as key themes, and the implementation of the strictest environmental protection system has received widespread attention. On January 1, 2018, the Environmental Protection Tax (EPT) Law of the People’s Republic of China was officially implemented. As one of the most representative economic measures in China’s pollution control efforts, the EPT policy aims to curb the fundamental trend of deteriorating environmental quality in China.

As a market-based environmental regulation tool, the EPT policy has significant economic and environmental effects. Scholars generally believe that the EPT policy can effectively reduce pollution emissions, promote economic development, increase public employment, and enhance social welfare. However, the well-being of the people is the starting point of “clear waters and green mountains,” and the health of the middle-aged and older adult is an important aspect of public welfare. Whether China’s implementation of environmental regulation policies, represented by the EPT system, can improve public health while controlling pollution, and how to enhance the health and well-being of the middle-aged and older adult, has become a critical area for assessment in the new era of environmental policies.

Since the implementation of the EPT policy, provincial governments have had the authority to set specific tax rates for air and water pollutants within the tax rate range, forming a dynamic adjustment mechanism where “the state sets the baseline, and local governments can adjust upwards.” Provinces such as Beijing, Tianjin, and Shandong have raised the pollutant tax rates to varying degrees. Generally, the government’s continuous promotion of ecological civilization, protection, and improvement of the environment, reduction of pollutant emissions, and increased public awareness and attention to environmental issues have facilitated positive public participation in environmental protection. This has helped reduce the exposure of middle-aged and older adult individuals to harmful substances, improving their psychological health, thereby lowering their disease incidence and reducing social medical costs.

Existing research primarily explores the effects of EPT from the perspective of firms or regions, focusing on aspects such as cleaner production, green technological innovation, and emission reduction effects. These studies provide crucial insights into the role of EPT in corporate production processes. Although there are studies addressing the health effects of EPT, most are based on macro models such as CGE models. These models typically emphasize the interactions between the overall economy and the environment rather than the direct experiences of individual residents. Individual residents are the direct beneficiaries or those affected by environmental policies. Examining the health effects of EPT from the perspective of individual residents allows for a more precise assessment of how the policy impacts people’s daily quality of life. This study aims to fill the existing research gap by investigating the health effects of EPT from the viewpoint of individual residents. This approach not only broadens the perspectives in EPT research but also provides policymakers with more detailed information for decision-making. Research at the individual level complements, rather than contradicts, macro-level research. While macro-level studies offer an overall evaluation of policy effects, individual-level studies deepen the understanding of these effects. Based on the findings from individual-level research, this study intends to propose more targeted policy recommendations to ensure that EPT policies more effectively enhance residents’ health outcomes.

Therefore, this paper takes the implementation of the EPT policy in 2018 as an external policy shock and selects cities that have raised the applicable tax rates for pollutants as the treatment group to study the health effects of tax reductions and environmental investment incentives. The contributions and innovations of this paper are mainly reflected in filling the gap in research on the health effects of the EPT policy from the perspective of individual residents. Existing analyses of the EPT policy mainly focus on the corporate or regional levels, with analytical variables primarily concentrated on environmental and economic benefits. This paper provides a more comprehensive evaluation of the effects of environmental regulation policies.

The rest of this paper is organized as follows: Section 2 reviews the literature on environmental pollution and public health; Section 3 introduces the theoretical basis for the impact of the EPT policy on the health of the middle-aged and older adult; Sections 4 and 5 report the model construction, variable settings, and empirical results; Section 6 discusses the path mechanisms from the perspectives of emission reduction effects and psychological effects; Section 7 conducts a heterogeneity analysis, revealing inter-group differences in urban and rural areas, age, and education levels; finally, Section 8 summarizes the paper and points out the limitations of existing research and future directions for improvement.

## 2 Literature review

Existing literature has reached a consensus on the harmful effects of environmental pollution on the physical and mental health of residents. Scholars in the fields of public health and medicine in China have repeatedly validated the relationship between environmental pollution and health levels through experimental and epidemiological analysis methods, evaluating the costs of air pollution (1). Physiologically, Currie and Neidell (2) and Deryugina et al. (3) found that air pollution induces pathological changes in multiple organs, leading to severe health issues such as cancer. The public bears heavier health costs, with rising mortality and morbidity rates. Chen et al. (4) found that environmental pollution is a significant factor affecting maternal mortality, with pollutants such as wastewater, exhaust gas, and solid waste likely to cause deadly conditions like internal complications, obstetric hemorrhage, and gestational hypertension. Based on the CHNS database, Tu et al. (5) discovered the negative impact of industrial nitrogen oxides on public health, where increased household income only slightly mitigates the harm of industrial dust. There is a significant correlation between environmental pollution exposure and adverse mental health effects (6). Due to environmental pollution, residents’ central nervous systems are damaged, and mental health issues, such as depression and lack of well-being, have become major problems (7). High levels of stress and anxiety disrupt the immune system, hindering physical health recovery (8). Perceptions of environmental quality affect fertility intentions through well-being and future expectations (9). Therefore, improving public health through pollution reduction and environmental protection has become a global consensus.

Existing studies have examined the pollution reduction, carbon reduction, and greening effects of the EPT from various perspectives, with relatively consistent conclusions (10). The EPT, characterized by its normative, mandatory, and authoritative nature, compels enterprises to adopt cleaner production practices and promotes green development within businesses (11), thereby increasing investment in environmental protection and the development of green technologies. As the implementation intensity of the EPT policy increases, enterprises, especially state-owned, heavily regulated, and financially constrained ones, are taking more initiative in assuming environmental social responsibility, significantly improving their environmental social responsibility scores (12). Based on micro-enterprise green patent data, Liu and Xiao (13) identified the green innovation incentive effect of the EPT, which has improved fossil energy usage efficiency and the ability to treat pollutants at the end of production processes. Using panel data from prefecture-level cities, Wang and Chen (14) found that the EPT has a significant emission reduction effect, lowering industrial sulfur dioxide emissions by 16.8%. The EPT policy has broken the “carbon curse” of resource-based cities, promoting industrial structure upgrades and green technological innovation in these cities (15). Scholars primarily use macro models such as CGE to estimate the health effects of the EPT. Wang et al. (16) measured that air pollution impacts national health and labor force loss, while energy taxes can reduce air pollutant concentrations. Xiao et al. (17) evaluated the effects of environmental taxes on health, education, and retirement using the OLG-DGE model, finding that environmental taxes improve individuals’ lifelong welfare.

Researching the impact of environmental regulation on the economy, health serves as an important link. However, the health effects of environmental regulation have been less frequently studied. Existing results indicate that environmental policies reduce health risks associated with environmental pollution (18). Zhang et al. (19), based on 976 environmental regulation policies from 1978 to 2013, found that environmental regulation policies promote public health. Do et al. (20) and Greenstone and Hanna (21) discovered that India’s Air Pollution Act effectively improved air quality, thereby reducing infant mortality rates. Tanaka (22), based on China’s acid rain control zones or sulfur dioxide pollution control zones delineated in 1998, found a 20% reduction in infant mortality. Xie and Feng (23) found a 3.3 to 4% reduction in morbidity among the working-age population, thus confirming the health effects of environmental regulation. Regarding China’s Air Pollution Prevention Action Plan (Ten Air Measures) in 2013, Fan et al. (24) found significant reductions in air and dust pollutants in the implementation areas, with notable health effects. Wang et al. (25) found that policies in green credit pilot zones improved resident health by enhancing the environment and public service levels, with this effect being more significant in western cities and resource-based cities.

In summary, environmental pollution reduces life expectancy and affects public health, making environmental regulation policies essential to mitigate the health hazards of environmental pollution. Although the economic and environmental effects of market-based environmental regulation policies, such as the EPT, have been extensively confirmed, the conclusions regarding their health effects remain unclear. It is imperative to further verify whether market-based environmental regulation policies can be as effective as punitive laws. Examining the impact of the EPT policy on public health and scientifically assessing the effectiveness of this policy will provide a reference for improving the EPT system and contribute to the reform of China’s tax system. Moreover, as the world’s largest developing country and the largest emitter of sulfur dioxide, China’s experience in promoting health through ecological governance has significant representativeness for developing countries in addressing environmental pollution.

## 3 Theoretical mechanism

The health of middle-aged and older adult individuals is influenced by various factors, including both internal and external elements. Among the external factors, the living environment is the most perceptible to the public and the most challenging to improve. Reducing the harmful impact of environmental pollution on public health has always been a pressing goal for government public sectors (23). As a representative component of China’s green tax system, the EPT aims at environmental protection and sustainable development. It embodies the polluter-pays principle and the green product incentive principle. By internalizing the costs of environmental pollution, the EPT encourages industrial enterprises to reduce pollution, promotes sustainable economic and environmental development, lowers public healthcare costs, and ultimately enhances the quality of life and health levels.

Based on Pigou’s theory of externalities, the negative externality of industrial pollution lies in the discrepancy between private and social costs borne by producers. Pollution generated during production and operations negatively affects the ecological environment and public health, impacts not reflected in the enterprises’ costs, hence representing a negative externality. Taxation is the optimal method to address negative externalities (26). The EPT addresses the challenge of environmental pollution’s negative externalities in a market economy by increasing the environmental burden on market entities during production and operations. Through the collaboration between environmental and tax authorities, the government can integrate tax information and pollution data, internalizing the external costs caused by enterprises to the ecological environment into their production and operation costs. This compels enterprises to consider environmental and health factors in their decision-making, thereby reducing the harmful effects of environmental pollution on public health, such as respiratory diseases caused by air pollution and digestive issues resulting from water pollution.

Residents’ life expectancy is jointly influenced by pollution stock and *per capita* output. The EPT, as a market-based environmental regulatory tool, links the taxation mechanism with pollutant emissions, embodying the principle of incentive compatibility and closely aligning with sustainable development goals. It emphasizes meeting current economic development needs without compromising the living environment for future generations (27). The implementation of the EPT can encourage enterprises to focus more on environmental protection and resource use efficiency, guiding societal resources away from high-energy, high-pollution industries toward green, low-carbon, and high-efficiency sectors. This promotes industrial structure optimization and upgrading, fostering coordinated development of economic growth and environmental protection. Through tax incentives and exemptions, the government can encourage technological innovation and management improvements in enterprises, driving the development of green technologies and products. A good environment is fundamental to maintaining public health. The implementation of the EPT helps enhance the nation’s capacity for environmental risk prevention and control, reducing public health issues caused by environmental pollution.

*Hypothesis 1*: As an economic policy tool, the EPT significantly enhances the health of middle-aged and older adult individuals.

An eco-friendly and livable environment provides a conducive condition for public health. Controlling the emission of pollutants harmful to human health is beneficial for improving public health (28). The EPT aids in encouraging enterprises to reduce pollutant emissions and improve environmental quality. Since polluters must bear the cost of environmental damage caused by their emissions, the EPT incentivizes industrial enterprises to reduce emissions, thereby lowering their tax burden. As a public good, the environment is characterized by non-exclusivity and non-competitiveness, making environmental resources susceptible to overuse and pollution. Through market mechanisms, the EPT stimulates enterprises to seek more environmentally friendly and efficient production methods and technologies. With the innovation and dissemination of pollution control technologies, urban enterprises are incentivized to adopt emission reduction measures, enhance energy efficiency, and transition to clean energy. This reduces greenhouse gas emissions and mitigates the adverse health impacts of climate change.

According to the “double dividend” hypothesis (29), the EPT can achieve coordinated economic growth and environmental protection. Urban resource allocation shifts toward more environmentally friendly and efficient directions, reducing the proportion of pollution-intensive industries and fostering the development of green industries. Therefore, the imposition of the EPT can decrease environmental pollutant emissions, improve environmental quality, and consequently lower societal environmental risks, positively impacting the health of middle-aged and older adult individuals. As an essential link for the coordinated development of environmental protection and economic growth, environmental regulatory pressure makes pollutant reduction imperative. According to the cost–benefit principle of the Porter Hypothesis, enterprises are compelled to adjust production modes, improve production processes, and upgrade pollution control equipment to prevent excessive energy consumption and inefficient resource allocation from the source. Furthermore, increasing the applicable tax rates for the EPT boosts local government fiscal revenues. This enhances the financial capacity of local governments to tackle environmental pollution, significantly improving local environmental quality.

*Hypothesis 2*: The EPT’s effectiveness in pollution control significantly impacts the health of middle-aged and older adult individuals through the “emission reduction effect.”

The World Health Organization (WHO) defines mental health as “a state of well-being in which the individual realizes his or her own abilities, can cope with the normal stresses of life, can work productively and fruitfully, and is able to make a contribution to his or her community.” Physical health refers to the normal functioning and health status of an individual’s body, including bodily functions, constitution, and immunity. The mutually reinforcing relationship between physical and mental health is bidirectional. Good mental health helps cultivate a positive lifestyle. Higher levels of happiness are associated with better physical health; optimistic emotions facilitate the body’s self-regulation, while stress and negative emotions can harm well-being and impact health and life expectancy (30–32). From a human capital perspective, individuals with a strong sense of well-being are more likely to adhere to better treatment plans, achieving higher health output efficiency (30). They also tend to lead healthier lifestyles (33), have better attitudes and perceptions toward health, and demonstrate higher efficiency in allocating health-related resources (34), such as quitting smoking and drinking, maintaining a balanced diet, and engaging in regular exercise.

The destruction of the natural environment can cause public psychological stress and anxiety, and the psychological state of middle-aged and older adult individuals significantly impacts their physical and mental health. For example, the risk of ozone-related depression significantly increases among people over 65 years old (35). The relationship between environmental protection and mental health originates from theories such as the biophilia hypothesis and psychological evolution. Humans and nature form a community of life; harmonious coexistence promotes immune system upgrades, regulates inflammatory responses, and provides numerous health benefits for humans (36). Given the close relationship between humans and the natural environment, which holds aesthetic and cultural value, ecological conservation is crucial for maintaining human psychological health (37).

On one hand, the EPT raises public awareness and consciousness about environmental protection, reducing instances of environmental injustice. This can enhance social trust and recognition, alleviate stress, reduce anxiety, and promote improved psychological health. On the other hand, increased environmental awareness and the widespread adoption of eco-friendly practices improve living environments. A beautiful, quiet, and clean living environment contributes to emotional stability, strengthens psychological resilience and coping abilities, and enhances psychological well-being and life satisfaction, thereby positively influencing mental health (38). Middle-aged and older adult individuals have needs for existence, relatedness, and growth (39). Middle-aged and older adult individuals have needs related to existence, relatedness, and growth (ERG needs). A clean environment can enhance social trust and recognition among these individuals, which is closely linked to their ERG needs. Enhanced social trust and recognition can fulfill their need for relatedness, while a clean environment provides better living conditions, addressing their need for existence. As these needs are met, their psychological well-being improves. The EPT policy is closely related to people’s aspirations for a better life. A clean environment gains the trust of middle-aged and older adult individuals, enhancing their social sense of belonging.

Therefore, the EPT may impact public health by improving sleep quality, fostering optimism, and enhancing overall happiness, which can be particularly beneficial for the health status of middle-aged and older adult individuals.

*Hypothesis 3*: The EPT significantly improves mental health, affecting the health of middle-aged and older adult individuals through the “psychological effect.”

## 4 Research design

### 4.1 Model construction

This study constructs a Difference-in-Differences (DID) model, using the implementation of the EPT as a quasi-natural experiment. The aim is to investigate the impact of increased pollutant tax rates and evaluate the policy effects of the EPT.

The specific model is expressed as follows:


(1) $$Health_{ijt}=\beta_{0}+\beta_{1}DID_{jt}+\gamma_{1}X_{it}+\gamma_{2}Z_{jt}+\mu_{j}+\nu_{t}+\varepsilon_{ijt}$$

Where: *i, j* and *t* represent individual samples, city samples, and years, respectively; $Health_{ijt}$ is the dependent variable, representing the health level of individual *i* in city *j* at time *t*; $DID_{jt}$ is the core explanatory variable of this study, with its coefficient $\beta_{1}$ reflecting the impact of the EPT policy on the health of middle-aged and older adult individuals; $X_{it}$ denotes individual-level control variables, including factors such as age, gender, marital status, and educational attainment; $Z_{jt}$ represents city-level control variables, including economic output, economic growth rate, the proportion of secondary industry, and the proportion of tertiary industry; $\mu_{j}$ represents the city fixed effect, $\nu_{t}$ represents the time fixed effect, and $\varepsilon_{ijt}$ is the random disturbance term.

### 4.2 Variable description

#### 4.2.1 Dependent variable

The dependent variable in this study is health level. Considering that self-rated health can accurately predict mortality risk and is highly correlated with other objective health indicators (40), this study primarily uses self-rated health to measure the health level of middle-aged and older adult individuals. According to the CHARLS questionnaire question “How would you rate your health status?,” responses of very poor, poor, fair, good, and very good are assigned values of 1, 2, 3, 4, and 5, respectively. In addition to self-rated health, this study constructs chronic disease status (Isdisease) as an alternative variable to examine the chronic conditions of middle-aged and older adult individuals, which refers to Ma et al. (41). According to the CHARLS question “Have you ever been told by a doctor that you have any of the following chronic diseases?” which includes 14 types of chronic diseases, responses are coded as 1 if the individual has any chronic disease and 0 otherwise.

#### 4.2.2 Core explanatory variable

The EPT policy imposes stricter requirements on local environmental pollution management. Within the tax rate table set by the national government, some regions have adjusted upward the tax rates on pollutant emissions. Following the methodology of Liu and Deng (10), this study categorizes cities with adjusted pollutant emission tax rates as the treatment group (treat = 1) and cities without adjustments as the control group (treat = 0). The variable time represents the implementation period of the EPT policy, with a value of 1 for 2018 and subsequent years, and 0 otherwise. The interaction of the group dummy variable (treat) and the policy implementation period dummy variable (time) constitutes the core explanatory variable DID in the model. Thus, individuals with DID = 1 represent those in regions where the pollutant emission tax rate was adjusted upward after the EPT policy implementation.

#### 4.2.3 Control variables

This study selects control variables from both urban and individual levels. At the urban level, considerations primarily encompass economic indicators such as total economic output (clngdp), economic growth rate (cggdp), the proportion of the secondary sector (cratio2), and the proportion of the tertiary sector (cratio3). At the individual level, the focus is on factors influencing resident health, primarily the living environment and household income. Urban economic scale and industrial structure reflect the economic and demographic characteristics of cities. Total economic output is represented by the logarithm of urban GDP, economic growth rate is denoted by the growth rate of urban GDP, and the proportions of the secondary and tertiary sectors are expressed as percentages of their respective output values. Individual-level variables mainly include gender (male), age (age), marital status (married), and level of education (educated). The rationale behind these selections lies in the understanding that advancing age may lead to declining physical function among the older adult, impacting their health. Additionally, variations in disease prevalence and health status among older adult individuals of different genders may exist. Stable marital relationships can provide psychological comfort and caregiving for the older adult, promoting their health. Differences in educational attainment may result in variations in occupational diseases among the older adult. The gender variable is coded as 1 for males and 0 otherwise; marital status is categorized based on survey responses, with individuals having a spouse coded as 1, including both those cohabiting with their spouses and those temporarily separated due to work or other reasons, and 0 otherwise; educational attainment ranges from no formal education to doctoral degree, with values assigned sequentially from 1 to 11 based on survey responses.

### 4.3 Data description

The data utilized in this study primarily stem from the China Health and Retirement Longitudinal Study (CHARLS). Initiated by Peking University, CHARLS surveys the health, economic well-being, and social participation of Chinese middle-aged and older adult individuals aged 45 and above, covering 150 counties and 450 communities across 28 provinces. Data have been publicly released for five waves spanning 2011, 2013, 2015, 2018, and 2020. This database provides robust support for formulating and refining policies to address population aging and meets the data demands for studying the health of middle-aged and older adult populations. Based on this foundation, individual-level survey data from the five waves are matched with corresponding urban-level data from the respective years, forming an unbalanced panel dataset of individual-urban pairs. Urban-level data primarily originate from the annual “China Urban Statistics Yearbook.”

### 4.4 Descriptive statistics

Table 1 presents descriptive statistics for the entire sample, with observations of chronic diseases excluding middle-aged and older adult individuals who did not complete the questionnaire. The mean value of self-rated health variables is 3.01, indicating that the average health status of Chinese middle-aged and older adult individuals is generally moderate. The 25th percentile is 2.00, suggesting that at least one-fourth of middle-aged and older adult individuals in China have a health status that is poor or below. Given that the variable for chronic diseases in this study is binary, with a mean value of 0.54, it implies that 54% of the surveyed middle-aged and older adult individuals suffer from chronic diseases. The mean values of the treatment group and post-policy variables are 0.68 and 0.23, respectively, indicating that 68% of the respondents reside in regions where the emission tax standard for pollution has been increased, and 23% of the periods are after the implementation of EPT policies. According to Table 1, the proportion of married middle-aged and older adult individuals in the observed sample is 87%, with a male proportion of 47%. The average age of middle-aged and older adult individuals is 60.15 years, with an average educational attainment level below completion of primary school. Looking at the data from the cities where the samples are located, the average proportion of the secondary sector is 40.59%, which is consistent with the national average; the average proportion of the tertiary sector is 38.55%, slightly lower than the national average. This discrepancy may be attributed to the fact that the survey scope of middle-aged and older adult individuals nationwide is determined by population proportion, and some populous provinces may not have well-developed tertiary sectors.

**Table 1:** Descriptive statistics.

Variable	Observations	Mean	Standard deviation	5th Quartile	25th Quartile	Median	75th Quartile	95th Quartile
Health	89,833	3.01	0.95	2.00	2.00	3.00	3.00	5.00
Isdisease	88,599	0.54	0.50	0.00	0.00	1.00	1.00	1.00
Treat	89,833	0.68	0.47	0.00	0.00	1.00	1.00	1.00
Time	89,833	0.23	0.42	0.00	0.00	0.00	0.00	1.00
Married	89,833	0.87	0.34	0.00	1.00	1.00	1.00	1.00
Male	89,833	0.47	0.50	0.00	0.00	0.00	1.00	1.00
Age	89,833	60.15	10.16	46.00	52.00	59.00	67.00	78.00
Educated	89,833	3.42	1.92	1.00	2.00	4.00	5.00	6.00
clngdp	89,833	7.57	0.96	6.03	6.99	7.48	8.11	9.39
cggdp	89,833	7.34	4.60	0.09	3.91	7.80	10.70	14.50
cratio2	89,833	40.59	15.76	0.41	36.28	44.28	50.36	59.67
cratio3	89,833	38.55	15.34	0.39	33.13	40.64	48.38	58.69

## 5 Analysis of empirical results

### 5.1 Benchmark regression results

To investigate whether EPT can improve the health status of middle-aged and older adult individuals, a double difference method is employed to evaluate the effect of policy implementation. The baseline regression results are presented in Table 2. The first, second, and third columns of Table 2 represent regression results without control variables, with individual characteristics as control variables, and with both individual and city characteristics as control variables, respectively. It is observed that the estimated coefficients of the key explanatory variable DID, the implementation of EPT, are significant at the 1% level, with estimated coefficients of 0.03 across all specifications. This indicates that after the implementation of EPT policies, in provinces where the emission tax for pollutants subject to EPT is increased, the self-rated health status of middle-aged and older adult individuals can improve by 0.03. Results in the third, fourth, and fifth columns of Table 2 demonstrate that after the implementation of EPT policies, in provinces where the emission tax for pollutants subject to EPT is increased, the prevalence of chronic diseases among middle-aged and older adult individuals can decrease by 0.03. This suggests that the imposition of EPT can effectively enhance individual health levels.

**Table 2:** Benchmark regression of the effect of EPT on the health of middle-aged and older adult.

Variable	Health			Isdisease		
	(1)	(2)	(3)	(4)	(5)	(6)
DID	0.0301*** (0.0110)	0.0300*** (0.0110)	0.0305*** (0.0113)	−0.0331*** (0.0078)	−0.0323*** (0.0078)	−0.0255*** (0.0079)
Married	–	0.0527** (0.0224)	0.0530** (0.0224)	–	0.0088 (0.0143)	0.0088 (0.0143)
Male	–	0.0689 (0.0923)	0.0706 (0.0923)	–	0.0135 (0.0512)	0.0120 (0.0512)
Age	–	0.0004 (0.0014)	0.0004 (0.0014)	–	0.0002 (0.0008)	0.0002 (0.0008)
Educated	–	0.0028 (0.0050)	0.0030 (0.0050)	–	0.0377*** (0.0036)	0.0375*** (0.0036)
clngdp	–	–	0.0290 (0.0252)	–	–	−0.0667*** (0.0150)
cggdp	–	–	0.0013 (0.0013)	–	–	0.0003 (0.0009)
cratio2	–	–	−0.0049** (0.0023)	–	–	0.0040*** (0.0015)
cratio3	–	–	−0.0057** (0.0023)	–	–	0.0019 (0.0015)
Individual fixed effects	Control	Control	Control	Control	Control	Control
Year fixed effects	Control	Control	Control	Control	Control	Control
Sample size	89,833	89,833	89,833	88,599	88,599	88,599
*R*-square	0.0009	0.0103	0.0003	0.0157	0.0058	0.0076

*, **, *** represent significance at the 10, 5, and 1% levels, respectively. Robust standard errors clustered at the individual level are reported, and subsequent tables follow the same format.

From the impact of EPT on the self-rated health and chronic diseases of middle-aged and older adult individuals as depicted in Table 2, it can be observed that the estimated coefficients of the key explanatory variable DID pass the 1% significance test, with estimated coefficients of 0.03 and −0.03, indicating that the increase in the EPT standard results in an increase of 0.03 units in self-rated health and a decrease of 0.03 units in the prevalence of chronic diseases. This implies that the imposition of EPT can significantly improve public health levels, promoting harmonious coexistence between humans and nature. In summary, the analysis suggests that the imposition of EPT can effectively enhance public health levels in areas where pollutant tax adjustments are made, thus validating hypothesis one.

### 5.2 Robust test

#### 5.2.1 Parallel trends test

Following Angrist and Pischke (42), this study employs a double difference method, necessitating an examination of whether the health levels of middle-aged and older adult individuals in areas subject to adjustments in pollution emission tax standards satisfy the parallel trends assumption compared to those in non-adjustment areas before the implementation of EPT policies. Drawing from Jacobson et al. (43), we construct the following model based on event study methodology to analyze dynamic effects, as shown in Equation 2.


(2) $$Health_{ijt}=\beta_{0}+\sum \theta_{t}Year_{t}*Treat_{i}+\rho_{1}X_{it}+\rho_{2}Z_{jt}+\gamma_{t}+\varphi_{i}+\varepsilon_{ijt}$$

**Figure 1:**
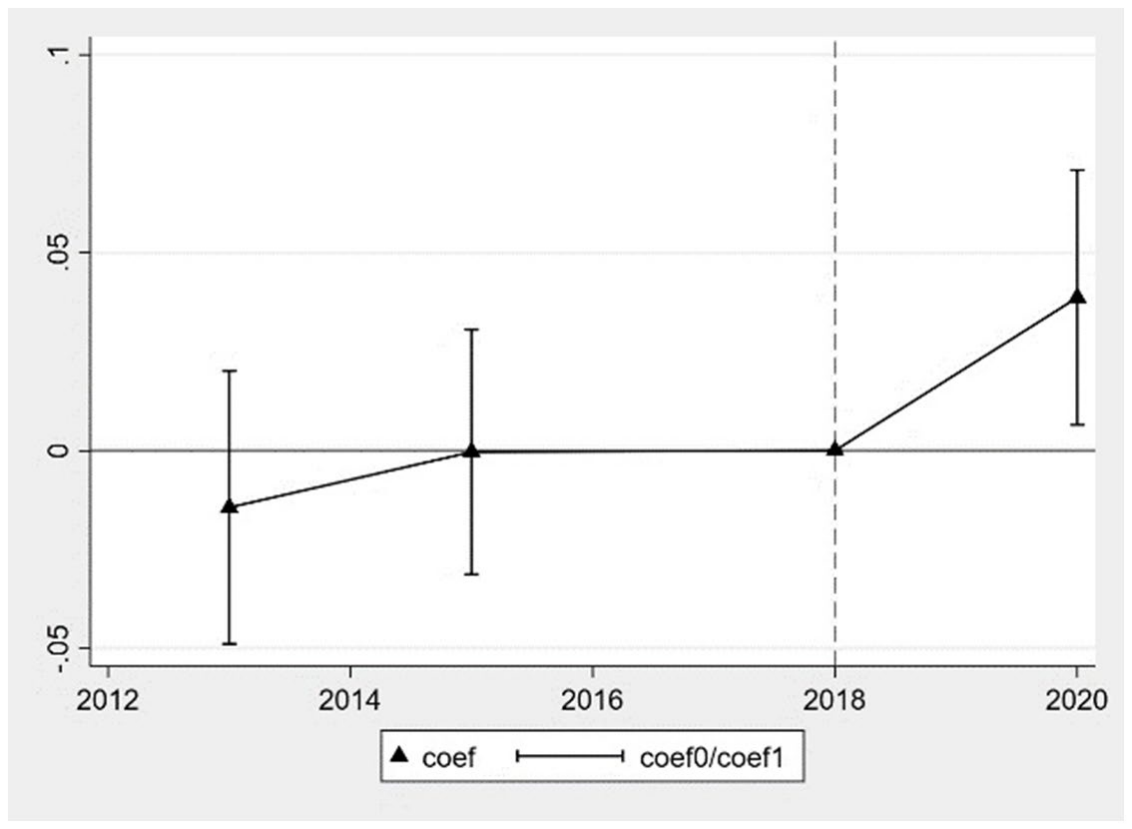
Parallel trends test for improvement in self-rated health.

**Figure 2:**
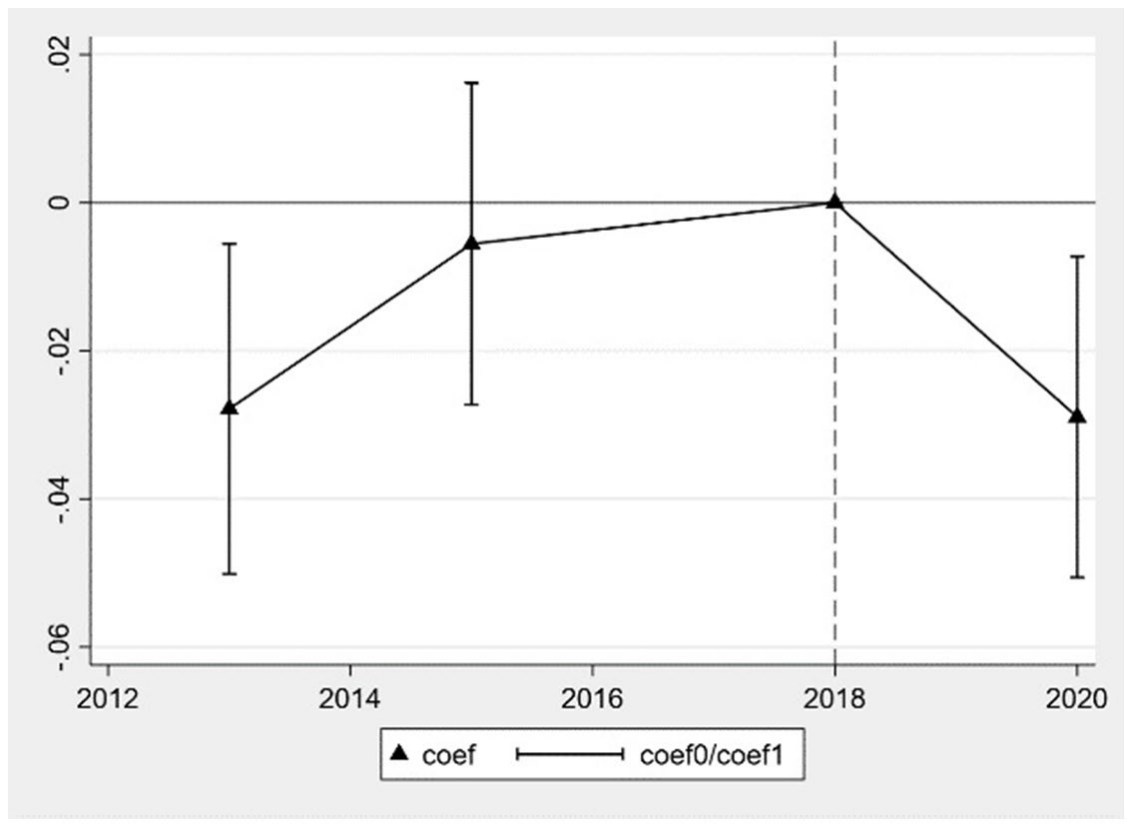
Parallel trends test for reduction in chronic diseases.

Equation 2 takes the year of implementation of EPT policies, 2018, as the base period and employs $Year_{t}$ as annual dummy variables, including the years 2013, 2015, and 2020. $\theta_{t}$ reflects the relative impact of the treatment group on health status in the t-th year, while other variables are defined as in Equation 1. According to the parallel trends assumption, the estimated coefficients of $\theta_{t}$ in 2013 and 2015 should not be significant, whereas they should be significantly different from zero in 2020. Utilizing Equation 2, the results are plotted as shown below, where coef represents the estimated coefficient of $\theta_{t}$ , and $coef_{0}$ and $coef_{1}$ represent the upper and lower bounds of the 95% confidence interval for $\theta_{t}$ , respectively. Figure 1 illustrates the parallel trends test for the improvement in self-rated health status, while Figure 2 depicts the parallel trends test for the reduction in chronic diseases. The results indicate that before the implementation of EPT policies, the estimated coefficients of the interaction term $Year_{t}*Treat_{i}$ for self-rated health and chronic diseases in 2013 and 2015 do not significantly differ from zero. This suggests that the health levels of the treatment and control groups satisfy the pre-treatment parallel trends assumption. After the implementation of EPT policies, the estimated coefficient of $\theta_{t}$ in 2020 is significantly different from zero. The lower bound of the confidence interval for the estimated coefficient of self-rated health is greater than zero, while the upper bound for the estimated coefficient of chronic diseases is less than zero, as depicted in the figure. This indicates that the implementation of EPT policies significantly improves the self-rated health status and reduces the prevalence of chronic diseases among middle-aged and older adult individuals. Thus, it is evident that the baseline regression meets the assumptions of the DID method, and the core explanatory variable “DID” in the baseline regression captures the health effects of EPT.

#### 5.2.2 Placebo test

**Figure 3:**
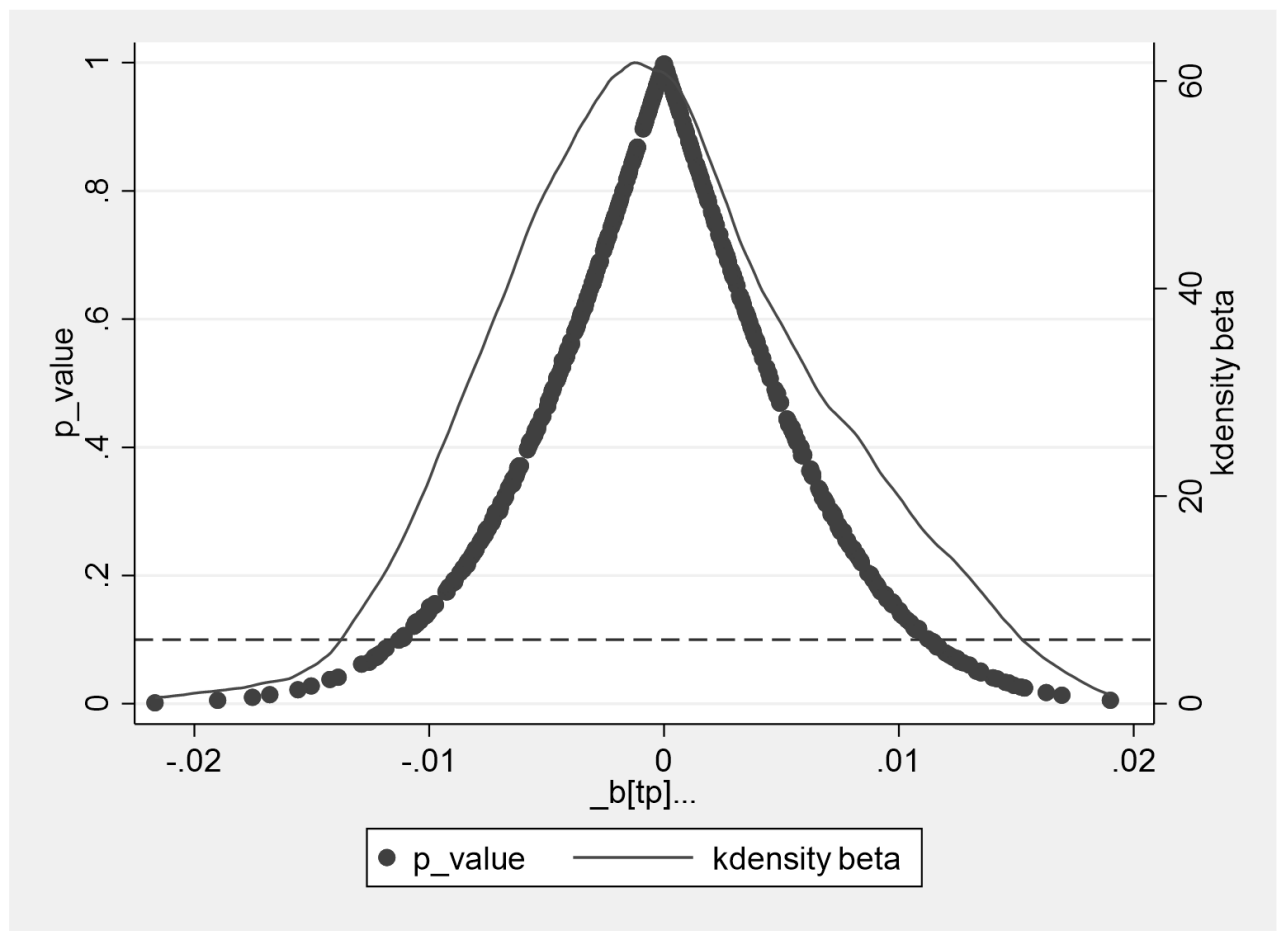
Placebo test for self-rated health.

**Figure 4:**
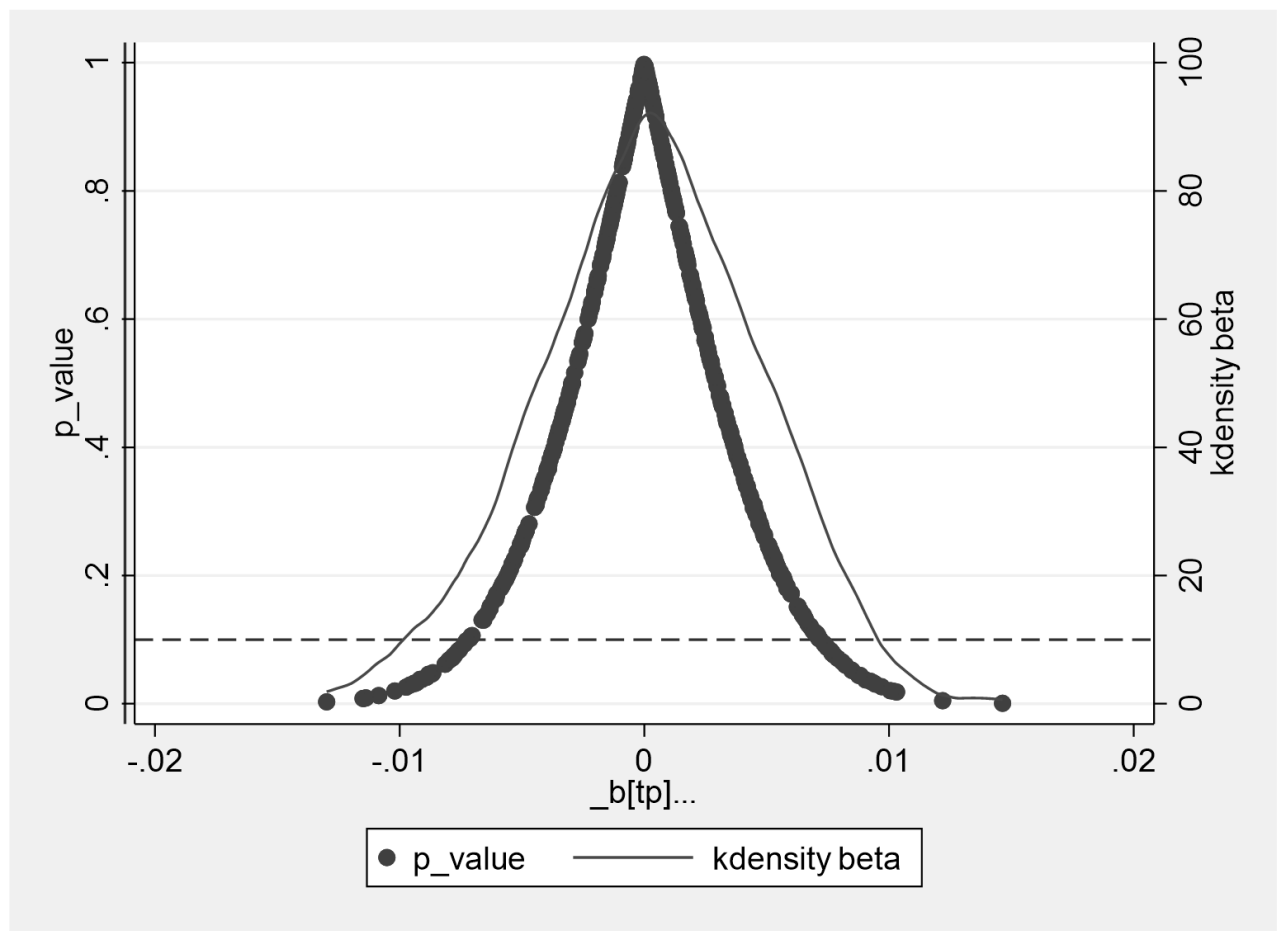
Placebo test for reduction in chronic diseases.

To address concerns regarding potential biases resulting from omitted key variables in the baseline regression, following the methodologies of Chetty et al. (44) and La Ferrara et al. (45), this study randomly draws 500 placebo treatment groups with the aim of employing the placebo test method to further validate the health effects of EPT. Through non-repeated random sampling, 500 virtual treatment groups for self-rated health and chronic diseases, respectively, are generated for the placebo test. Consequently, 500 virtual DID estimates are obtained, with “_b[tp]” listed on the *x*-axis of Figures 3, 4, represented by solid circular points. In Figures 3, 4, the left and right *y*-axes, respectively, represent the *p*-values of virtual DID estimates and their kernel density distributions. It can be observed clearly that the estimated coefficients of virtual DID follow a normal distribution, consistent with the expected outcomes of the placebo test. Furthermore, in the baseline regression testing self-rated health status, the actual coefficient of the explanatory variable DID is 0.0305 [see Table 2, column (3)], significantly larger than the simulated counterfactual estimate. Conversely, in the baseline regression examining the prevalence of chronic diseases, the actual coefficient of the explanatory variable DID is −0.0255 [see Table 2, column (6)], significantly smaller than the simulated counterfactual estimate. The placebo results corroborate the health effects of EPT from a counterfactual perspective and are less influenced by potential unobservable factors.

#### 5.2.3 PSM-DID test

To address potential systematic differences in the selection of treatment and control groups, this study employs the Propensity Score Matching-Difference-in-Differences (PSM-DID) method to further validate the health effects of EPT, aiming to minimize the influence of regional economic factors and individual characteristics. Utilizing control variables as identifying features for sample individuals, suitable control group samples are selected for older adult individuals in the treatment group through 1:1 nearest neighbor matching. Subsequently, the coefficient of the core explanatory variable DID is estimated using the matched samples through the DID method. The regression results after matching are presented in Table 3. Overall, the estimated coefficient of DID remains significant. In the first column of Table 3, the coefficient of 0.0403 is significant at the 5% confidence level, slightly larger in magnitude than the 0.0305 in the baseline regression [Table 2, column (3)]. In the second column of Table 3, the coefficient of −0.0305 is significant at the 1% confidence level, slightly smaller in magnitude than the −0.0255 in the baseline regression [Table 2, column (6)]. This result indicates that the conclusion drawn from the baseline regression is further reinforced by the PSM-DID test, suggesting that the implementation of EPT policies enhances the health status of middle-aged and older adult individuals in the matched sample data.

**Table 3:** PSM-DID test.

Variable	Health	Isdisease
	(1)	(2)
DID	0.0403** (0.0168)	−0.0305*** (0.0115)
Married	0.0476 (0.0321)	0.0216 (0.0199)
Male	0.1052 (0.1280)	−0.0017 (0.0812)
Age	−0.0007 (0.0022)	0.0004 (0.0012)
Educated	0.0081 (0.0073)	0.0389*** (0.0051)
clngdp	0.0830** (0.0418)	−0.0900 (0.0277)
cggdp	0.0003 (0.0018)	0.0010 (0.0012)
cratio2	−0.0057* (0.0034)	0.0052** (0.0021)
cratio3	−0.0039 (0.0033)	0.0031 (0.0021)
Individual fixed effects	Control	Control
Year fixed effects	Control	Control
Sample size	52,401	51,800
*R*-square	0.0024	0.0049

#### 5.2.4 Exclusion of other policy interference

During the window period from 2011 to 2020, in addition to the EPT policy, other environmental protection policies were also implemented. These policies may have influenced the environment and subsequently impacted the health of middle-aged and older adult individuals through environmental pathways. Among these, the creation policy of China’s new energy demonstration cities is particularly relevant to environmental protection and residents’ lives. This policy has recently garnered attention from scholars (46–48). Their findings suggest that energy transition pressures have prompted industrial enterprises to innovate toward green practices, thereby promoting the green and low-carbon development of demonstration cities. The list of China’s new energy demonstration cities was released by the National Energy Administration in 2014, aiming to facilitate the green transformation of China’s energy development. The list includes 81 new energy demonstration cities.

This study considers the potential interaction between the EPT policy and the policy of constructing new energy demonstration cities during the sample period, which may have interfered with the health effects of the EPT policy. Following the approach of Li et al. (49), a policy dummy variable DID2 representing new energy demonstration cities is constructed and added to Equation 1 of the baseline regression model as a control variable. The regression results are presented in Table 4. Observing the results in the first and second columns of Table 4, it is found that the regression coefficients of the core explanatory variable DID are significant at the 1% confidence level, and their magnitudes do not differ from those in the baseline regression [Table 2, column (3) and column (6)]. This result validates the robustness of the empirical conclusions of this study.

**Table 4:** Exclusion of policy interference from new energy demonstration zones.

Variable	Health	Isdisease
	(1)	(2)
DID	0.0305*** (0.0113)	−0.0255*** (0.0079)
DID2	−0.0087 (0.0176)	0.0133 (0.0091)
Married	0.0529** (0.0224)	0.0088 (0.0143)
Male	0.0704 (0.0923)	0.0123 (0.0513)
Age	0.0004 (0.0014)	0.0002 (0.0008)
Educated	0.0030 (0.0050)	0.0375*** (0.0036)
clngdp	0.0294 (0.0253)	−0.0673*** (0.0150)
cggdp	0.0013 (0.0013)	0.0003 (0.0009)
cratio2	−0.0049** (0.0023)	0.0040*** (0.0015)
cratio3	−0.0057** (0.0023)	0.0019 (0.0015)
Individual fixed effects	Control	Control
Year fixed effects	Control	Control
Sample size	89,833	51,800
*R*-square	0.0003	0.0075

Furthermore, as the policy of constructing new energy demonstration cities primarily serves as an incentive-based policy, while the EPT policy is predominantly punitive in nature, it is observed that the coefficients of the core explanatory variable DID for the EPT policy are significantly positive or negative, whereas the virtual explanatory variable DID2 for the new energy demonstration city construction policy is not significant. This indicates that tax policies with punitive or mandatory characteristics have a more tangible effect, and the EPT policy more directly improves the health status of middle-aged and older adult individuals.

#### 5.2.5 The relationship between self-rated health and expected future survival probability

Due to the potential mixture of randomness and subjectivity in respondents’ answers to self-rated health levels, this study employs objective responses regarding chronic diseases to supplement the assessment of middle-aged and older adult individuals’ health status. Additionally, we assess the correlation between respondents’ self-rated health and their expected future survival probability. If respondents answer the survey questions more seriously, their responses to questions with similar implications should be more consistent. Thus, the self-rated health variable should be more correlated with the expected future survival probability. Expected future survival probability reflects subjective life expectancy. In the Chinese CHARLS questionnaire, respondents are first grouped into different age ranges, and then asked about the likelihood of reaching a target age, which is approximately 10–15 years older than their actual age at the time of response (Groups A, B, C, D, E, F, G, H, and I; corresponding to ages less than 65, 65–69, 70–74, 75–79, 80–84, 85–89, 90–94, 95–99, and greater than or equal to 100; and target ages of 75, 80, 85, 90, 95, 100, 105, 110, and 115, respectively). Valid responses range from 1 to 5, representing almost impossible, unlikely, possible, likely, and almost certain, respectively. Since age is controlled for as a variable, there is no need to be concerned about its direct impact on respondents’ expectations of future survival probability. Table 5 presents the correlation between self-rated health and expected future survival probability. The first column does not include control variables, while the second column includes individual and urban factors. The results indicate a high consistency between self-rated health levels and respondents’ expected future survival probability, both significantly positive at the 1% level, suggesting a certain level of credibility in the self-rated health level, one of the explanatory variables. Additionally, the coefficient of the age variable in the second column of Table 5 is significantly negative at the 1% confidence level, indicating that as middle-aged and older adult individuals age, they have a clear awareness of approaching life expectancy, consistent with common sense.

**Table 5:** Relationship between self-rated health and expected future survival probability.

Variable	(1)	(2)
Health	0.1883*** (0.0073)	0.1879*** (0.0073)
Married	–	0.1161*** (0.0357)
Male	–	0.1352 (0.1379)
Age	–	−0.0099*** (0.0024)
Educated	–	0.0124* (0.0073)
clngdp	–	−0.0371 (0.0323)
cggdp	–	0.0005 (0.0019)
cratio2	–	−0.0012 (0.0032)
cratio3	–	−0.0017 (0.0032)
Individual fixed effects	Control	Control
Year fixed effects	Control	Control
Sample size	74,282	74,282
*R*-square	0.1279	0.1277

## 6 Mechanism test

Based on the theoretical analysis presented earlier, the EPT policy may enhance the health status of middle-aged and older adult individuals through two channels: the “emission reduction effect” and the “psychological effect.” The following sections of this paper examine the pathway mechanisms of the health effects of the EPT through these two channels.

### 6.1 Emission reduction effect of EPT

As an economic instrument, the EPT policy incentivizes enterprises and individuals to reduce emissions of pollutants, aiming to improve the environmental quality upon which public life depends. By increasing operational costs for enterprises, the EPT policy stimulates enterprises to seek methods to reduce pollutant emissions. Enterprises may adopt advanced technologies and production equipment, optimize and update production processes, and reduce emissions of pollutants such as exhaust gasses and dust during production to achieve the goal of reducing tax payments. Additionally, the EPT policy promotes transparency in corporate pollution information, drawing close attention from local governments. Due to the pressure faced by Chinese government officials in “political competitions,” where green environmental protection is an important component of performance assessments, government departments may exert pressure on high-polluting and high-emission enterprises, driving these companies to undergo green transformation or even closing down some irreparable polluting enterprises. With increased tax revenue, government departments have more funds available for environmental pollution control and new environmental protection projects, further improving environmental quality through investment in environmental projects.

According to the “Environmental Protection Tax Law of the People’s Republic of China,” taxable pollutants include atmospheric pollutants, water pollutants, solid pollutants, and noise, among others, among which atmospheric pollutants are subject to significant changes over time and space, making them susceptible to the influence of EPT policies. Therefore, this study focuses on observing changes in atmospheric pollutant concentrations in various cities in the pathway mechanism examination. In recent years, Chinese residents have been particularly sensitive to particulate matter (PM) and sulfur dioxide (SO2) pollution in atmospheric pollutants. When the concentration of such atmospheric pollutants reaches harmful levels, the conditions for normal public living and development are disrupted. This study selects PM10 and PM2.5, which are widely concerned by the public, as representatives of particulate matter pollution, and sulfur dioxide as a representative of sulfur oxide pollution, to examine the impact of EPT policies on the reduction of regional atmospheric pollutant emissions. The concentrations of PM10, PM2.5, and sulfur dioxide in prefecture-level cities are sourced from the “China City Air Quality Index” of the Guotai An database, measured in micrograms per cubic meter. The time window selected is from 2013 to 2022 to facilitate the organization and standardization of monthly data. Both PM10 and PM2.5 can remain suspended in the atmosphere for a long time and contain toxic and harmful substances, and their damage to human health has become an indisputable fact.

Table 6 presents the regression results of the EPT policy on the concentrations of PM10, PM2.5, and sulfur dioxide (SO2), both with and without control variables. The estimated coefficients of the core explanatory variable, DID, are negative in columns one through six of Table 6. Specifically, in columns one and two, the estimated coefficients are significant at the 5% confidence level, with approximate values of −2.5 without control variables and −2.4 with control variables. However, in columns three and four, although the estimated coefficients of DID are negative, they are not significant, and the absolute values of the coefficients are much smaller than those of the regression coefficients for PM10 concentration. This result indicates that after the implementation of the EPT policy, there is a decrease in both PM10 and PM2.5 concentrations in regions with increased pollution emission tax collection standards. However, the EPT policy has a more direct impact on PM10 concentration reduction, while reducing PM2.5 concentration poses some difficulty. This aligns with empirical findings and common intuition, as PM10 particles, with a diameter equal to or greater than 10 micrometers, settle more easily than PM2.5 particles, which have a smaller diameter. Therefore, the implementation of the EPT policy primarily reduces the concentration of PM10 particles that are more prone to settling, while the short-term effect on reducing PM2.5 concentration is less significant.

**Table 6:** Impact of EPT on air quality.

Variable	PM10	PM10	PM2.5	PM2.5	LNSO2	LNSO2
	(1)	(2)	(3)	(4)	(5)	(6)
DID	−2.5464** (1.2313)	−2.3676** (1.2001)	−0.8404 (0.8211)	−0.6368 (0.8147)	−0.0561* (0.0290)	−0.0517* (0.03001)
clngdp	–	7.2268** (2.8030)	–	0.5737 (1.9270)	–	−0.0258 (0.0655)
cggdp	–	0.0145 (0.0376)	–	0.0299 (0.0407)	–	0.0000 (0.0006)
cratio2	–	0.0035* (0.0623)	–	0.0225 (0.0434)	–	−0.0026** (0.0013)
cratio3	–	−0.246*** (0.0573)	–	−0.1490*** (0.0374)	–	−0.0005 (0.00127)
Individual fixed effects	Control	Control	Control	Control	Control	Control
Year fixed effects	Control	Control	Control	Control	Control	Control
Sample size	30,799	28,969	30,799	28,969	30,799	28,969
*R*-square	0.3925	0.3981	0.0857	0.0853	0.4463	0.4384

Furthermore, the results in columns five and six indicate that the coefficients of the core explanatory variable, DID, pass the 10% significance test, with an estimated value of approximately −0.05. This suggests that after the implementation of the EPT policy, compared to regions without adjustments, sulfur dioxide emissions can be reduced by around 5% with adjusted tax rates. This demonstrates the effective role of the EPT policy in reducing emissions, lowering energy consumption and pollution emissions of social enterprises, directly reducing pollutant levels in the air, improving regional air quality, and thereby safeguarding the health of middle-aged and older adult individuals in the area.

### 6.2 Psychological effects of EPT

Environmental Protection Tax, as an economic instrument targeting environmental pollution, has a significant promoting effect on emission reduction and environmental protection by enterprises. However, due to its subtle impact on residents’ psychological well-being, it is often overlooked. The Environmental Protection Tax Law is China’s first single-line tax law specifically reflecting “green taxation” and has garnered widespread attention from various sectors of society. Through important promotional activities such as Tax Promotion Day, World Environment Day, and National Ecology Day, EPT policies have been popularized nationwide, enhancing public awareness of environmental protection and providing positive incentives to the public. Psychological health is closely related to physical health. This paper believes that the psychological effects of EPT policies on the public, especially middle-aged and older adult individuals, mainly manifest in two aspects: direct psychological effects and indirect psychological effects. Direct psychological effects refer to the impact directly on the psychological well-being of middle-aged and older adult individuals, while indirect psychological effects refer to the psychological impact caused by media dissemination and public searches.

#### 6.2.1 Direct psychological effect

This paper uses sleep quality and memory condition as proxy variables for direct psychological conditions to study the direct psychological reactions of middle-aged and older adult individuals. The biological clock is an important mechanism regulating human sleep. Psychological health problems such as anxiety, depression, and stress make it difficult for individuals to relax and fall asleep, directly affecting their sleep quality. The method of using sleep quality to measure psychological health is partially based on the research of Xue and Ge (50) and has been modified based on the CHARLS questionnaire. Data is sourced from respondents’ answers to the question “the average actual sleep time per night in the past month,” which highlights the “actual sleep time,” excluding the interference time of “lying in bed but not sleeping or unable to sleep.” Longer actual sleep time indicates better sleep quality. Additionally, psychological health is closely related to memory condition. When psychological health is good, individuals are more likely to remember important information and experiences because psychological health helps individuals better regulate their emotions and mental states, avoiding negative effects on memory due to emotional fluctuations or anxiety. Therefore, using memory condition as a proxy variable for individual psychological health has scientific validity. Data is sourced from respondents’ answers to the question “how do you rate your current memory condition,” where the five levels of response from 1 to 5 represent excellent, very good, good, fair, and poor, respectively. Thus, smaller numerical values indicate better memory condition.

Based on the preceding analysis, EPT can easily influence residents’ behavior and psychology, thereby altering the health status of middle-aged and older adult individuals. The dependent variables in columns one and two of Table 7 are sleep quality and memory condition, respectively. It is found that the coefficients of the core explanatory variable, DID, are significant at the 5 and 10% confidence levels, with estimated coefficients of 0.0495 and −0.0167, respectively.

**Table 7:** The impact of EPT on psychological health.

Variable	Sleep quality	Memory condition
	(1)	(2)
DID	0.0495** (0.0243)	−0.0167* (0.0099)
Married	0.3263*** (0.0556)	0.0178 (0.0207)
Male	0.1219 (0.1879)	−0.0856 (0.0843)
Age	−0.0035 (0.0039)	0.0003 (0.0012)
Educated	−0.0098 (0.0108)	0.0086* (0.0047)
clngdp	0.0564 (0.0494)	−0.0418 (0.0225)
cggdp	−0.0056** (0.0028)	−0.0004 (0.0011)
cratio2	−0.0089** (0.0050)	0.0015 (0.0020)
cratio3	−0.0130*** (0.0050)	0.0015 (0.0020)
Individual fixed effects	Control	Control
Year fixed effects	Control	Control
Sample size	89,692	89,288
*R*-square	0.0038	0.0002

The above results indicate that after the implementation of EPT policies, areas where pollution emission tax standards have been raised show an increase in sleep duration among middle-aged and older adult individuals, leading to improved sleep quality and memory, thereby significantly enhancing their psychological health status. Furthermore, among the controlled variables, the coefficient of the marital status variable on sleep quality is 0.3263, significant at the 1% level, consistent with previous research. While the common understanding that sleep quality deteriorates with age seems to be reflected in this study, it is not significant. Therefore, EPT policies may have a potential impact on the psychological health of middle-aged and older adult individuals. Firstly, they serve a psychological suggestive role, and secondly, they contribute to environmental improvement. These factors collectively contribute to enhancing the quality of life for middle-aged and older adult individuals, thereby safeguarding their physical and mental health.

#### 6.2.2 Indirect psychological effect

In this study, environmental awareness is utilized as a proxy variable for indirect psychological effects, encompassing the media indices and mobile device search indices of environmental pollution and haze, all obtained from Baidu Index. Prior research has often employed Baidu Index to measure variables such as investor sentiment, industry attention, and investor interest (51). In this context, regional indices related to environmental issues are employed to measure residents’ environmental concern, indirectly reflecting their psychological responses. It is well known that news media serves as a primary channel for citizens to acquire information and understand the world, and the content of news media affects individuals’ psychological health. The media index reflects the extent to which citizens passively receive information from media platforms, but due to differences in information processing abilities, it cannot fully represent citizens’ own emotions. Conversely, mobile device search indices primarily reflect citizens’ subjective initiative, with fluctuations in search indices being closely related to the zeitgeist and individual concerns. By examining the impact of EPT policies on news media and search indices, this study indirectly explores changes in citizens’ psychological activities and thereby verifies the indirect psychological effects of EPT.

The EPT policies are closely related to public life, and related topics often become the focus of local media. Table 8 describes the impact of the implementation of EPT on environmental indices of media and search. The first and second columns depict the effects on relevant news and search indices related to “environmental pollution,” while the third and fourth columns describe the effects on haze-related news and search indices. According to the results in the first and third columns, following the implementation of EPT policies, media coverage of “environmental pollution” and “haze” increases with the rise in pollution emission tax standards. Since haze is one of the conditions caused by environmental pollution, the effect of reporting on environmental pollution is more pronounced. Therefore, environmental pollution tax policies promote regional attention to environmental issues, with local media paying more attention to news related to environmental pollution.

**Table 8:** The impact of EPT on search indices.

Variable	Environmental pollution news media index	Environmental pollution mobile search index	Haze news media index	Haze mobile search index
	(1)	(2)	(3)	(4)
DID	26926.5100** (10000.0200)	4.2881* (2.2223)	1780.9260** (784.3900)	−18.6843** (8.2486)
clngdp	2907.6140 (32227.0800)	0.1724 (7.5159)	1166.6100 (2767.4240)	−12.5509 (24.7127)
cggdp	−7840.2640 (28667.5400)	36.0981** (13.2748)	−1485.1920 (2384.1560)	197.6398** (89.4861)
cratio2	885.5074 (3572.8800)	−0.1208 (1.0670)	32.8119 (341.4503)	−2.0812 (2.6081)
cratio3	294.0568 (2935.7710)	0.5919 (0.7318)	−49.9846 (274.1452)	−1.1848 (2.1235)
Individual fixed effects	Control	Control	Control	Control
Year fixed effects	Control	Control	Control	Control
Sample size	186	372	186	372
*R*-square	0.4920	0.6328	0.5346	0.2278

However, there is a discrepancy between the results of the second and fourth columns. The estimated coefficient of the core explanatory variable DID in the second column is 4.2881, significant at the 10% confidence level, while in the fourth column, the coefficient is −18.6843, significant at the 5% confidence level, with the two coefficients being opposite. The implementation of EPT policies leads to an increase in mobile device search volume for environmental pollution and a decrease in search volume for haze, indicating an increase in residents’ attention to environmental pollution but a decrease in their attention to haze. The reason for this result may be that after the implementation of EPT, influenced by government propaganda and media coverage, residents are more likely to focus on environmental pollution and actively search for news related to environmental pollution. At the same time, the air quality in the region may improve, leading to a significant decrease in residents’ searches for haze-related information. Psychological factors, as reflected through news media dissemination and mobile device searches, demonstrate the effects of environmental pollution tax policies, indicating an enhancement in residents’ psychological resilience and attention in regions where pollution tax standards are raised.

## 7 Heterogeneity test

### 7.1 Urban and rural

According to the theory of environmental equity, the impact of environmental pollution on the health levels of different social groups varies. Under the same environmental policy, the effects of health improvement among different social groups may exhibit significant disparities. By analyzing the characteristics of different groups of older adult individuals in urban and rural areas, we can capture the underlying mechanisms of the impact of EPT on health levels and thereby identify public groups sensitive to environmental regulatory policies. From the perspective of the environmental transmission mechanism, since the main subjects of EPT collection are pollution-intensive enterprises, and most of China’s enterprises are located in urban development zones, severe industrial emissions and noise pollution issues have arisen. In contrast, rural soil and water pollution mainly stem from agricultural production activities, which are less affected by EPT. Additionally, rural areas boast higher levels of green coverage compared to urban areas, with rich natural ecological environments. However, from the perspective of the psychological transmission mechanism, due to higher levels of education and information acquisition capabilities among urban residents compared to rural residents, urban residents possess a stronger environmental awareness. Consequently, at the psychological perception level, urban residents exhibit greater sensitivity to environmental improvements, thereby increasing the likelihood of psychological well-being improvements and subsequently enhancing health levels.

Table 9 reflects changes in the health levels of urban and rural older adult individuals following the implementation of EPT. The first and second columns, respectively, indicate the effects on the prevalence of chronic diseases and self-rated health among urban older adult individuals following the implementation of EPT, while the third and fourth columns depict the effects on the prevalence of chronic diseases and self-rated health among rural older adult individuals. The regression process includes control variables for individual characteristics and urban features. Overall, EPT leads to a decrease in the prevalence of chronic diseases and an improvement in the self-rated health levels of the older adult. However, from the analysis of significance, it is evident that the estimated coefficient of the core explanatory variable DID in the second column is significant at the 1% level, with a coefficient of 0.0618, indicating a significant improvement in the self-rated health levels of urban residents due to EPT. In contrast, the estimated coefficient of DID for self-rated health among rural older adult individuals in the fourth column is not significant. Since self-rated health is closely related to psychological perception channels, this finding further corroborates the psychological channels through which EPT enhance the health levels of the older adult.

**Table 9:** Urban–rural disparities.

Variable	Urban		Rural	
	Isdisease	Health	Isdisease	Health
	(1)	(2)	(3)	(4)
DID	−0.0176 (0.0128)	0.0618*** (0.0173)	−0.0303*** (0.0101)	0.0135 (0.0149)
Married	0.0188 (0.0223)	0.0883** (0.0350)	0.0022 (0.0186)	0.0297 (0.0291)
Male	−0.0107 (0.0939)	0.2431* (0.1391)	0.0298 (0.0604)	−0.0156 (0.1183)
Age	0.0023 (0.0015)	0.0085 (0.0031)	−0.0008 (0.0009)	0.0009 (0.0015)
Educated	0.0574*** (0.0052)	0.0013 (0.0070)	0.0182*** (0.0050)	0.0052 (0.0071)
clngdp	−0.0728*** (0.0267)	0.0437 (0.0390)	−0.0674*** (0.0182)	0.0112 (0.0327)
cggdp	−0.0010 (0.0015)	0.0042* (0.0022)	0.0017 (0.0011)	−0.0006 (0.0016)
cratio2	0.0049* (0.0026)	0.0048 (0.0036)	0.0047** (0.0018)	−0.0052* (0.0029)
cratio3	0.0000 (0.0025)	−0.0021 (0.0036)	0.0042 (0.0019)	−0.0082*** (0.0030)
Individual fixed effects	Control	Control	Control	Control
Year fixed effects	Control	Control	Control	Control
Sample size	35,166	35,748	53,433	54,085
*R*-square	0.0095	0.0081	0.0055	0.0043

On the other hand, the estimated coefficient of DID in the third column is significantly negative at the 1% level, with a coefficient of −0.0303, indicating a significant decrease in the prevalence of chronic diseases among rural older adult individuals following the implementation of EPT. In contrast, the coefficient of DID in the first column is not significant, implying that in terms of actual physical health, the implementation of EPT significantly improves the health levels of older adult individuals in rural communities through environmental protection channels. This may be due to inadequate levels of medical care and prevention awareness among rural residents. The environment, as an exogenous variable, is crucial for the prevalence of chronic diseases among rural older adult individuals. Therefore, following the implementation of EPT, the chronic disease status of rural older adult individuals significantly improves.

### 7.2 Middle-aged and older adult

The implementation of EPT has varying effects on middle-aged and older adult individuals. In China, the typical retirement age for women is 55, and for men, it is 60. According to international standards, individuals aged 60 and above are considered older adult. Therefore, this study divides the middle-aged and older adult population into two categories: those under 60 and those aged 60 and above. Generally, older adult individuals experience a decline in physical function and may be more sensitive to harmful substances in the environment, making them more susceptible to the effects of environmental pollution. Clean, safe, and peaceful living environments are crucial for the quality of life of the older adult and contribute to improving their mental health. However, middle-aged individuals still face pressures related to raising children and caring for older adult parents. Instances of psychological burdens and stress among middle-aged individuals are not uncommon in China. A pleasant environment can alleviate these situations through psychological channels. Therefore, if EPT improve the health levels of middle-aged and older adult individuals through environmental channels, the coefficients should be more significant among the middle-aged and older adult population. Conversely, if they improve health levels through psychological channels, the coefficients should be more significant among the middle-aged population. Studies have found that EPT have a more significant effect on the physical and mental health of older adult individuals, as long-term environmental pollution significantly exacerbates chronic disease symptoms among the older adult.

According to Table 10, the first and second columns, respectively, indicate the effects of EPT on the prevalence of chronic diseases and self-rated health among the older adult, while the third and fourth columns represent changes in the prevalence of chronic diseases and self-rated health among middle-aged individuals following the implementation of EPT. Overall, EPT has effectively reduced the prevalence of chronic diseases in both groups and improved the self-rated health of middle-aged and older adult individuals. However, there is a considerable difference in the significance between the two groups, with a higher significance observed among the older adult population. The coefficient of the main explanatory variable DID in the first column of Table 10 is significant at the 1% level, with a coefficient of −0.03, indicating a more significant improvement in the chronic disease status of the older adult due to the implementation of EPT. Furthermore, the coefficient of DID in the second column of Table 10 is significant at the 5% level, with a coefficient of 0.0395, while the coefficient of DID in the fourth column is not significant, indicating that the implementation of EPT policies significantly improves the self-rated health of the older adult. Whether through psychological or environmental channels, the older adult benefit more significantly from EPT policies.

**Table 10:** Age differences.

Variable	60 and above		Less than 60	
	Isdisease	Health	Isdisease	Health
	(1)	(2)	(3)	(4)
DID	−0.0300*** (0.0112)	0.0395** (0.0167)	−0.0201* (0.0118)	0.0232 (0.0166)
Married	−0.0213 (0.0171)	0.0402 (0.0277)	−0.0047 (0.0293)	0.0339 (0.0412)
Male	0.0000 (0.0787)	−0.0680 (0.1412)	0.0045 (0.0737)	0.1710 (0.1409)
Age	−0.0009 (0.0013)	0.0006 (0.0025)	0.0045 (0.0033)	−0.0039 (0.0044)
Educated	0.0019 (0.0061)	−0.0065 (0.0081)	0.0574*** (0.0047)	0.0041 (0.0069)
clngdp	−0.0664*** (0.0206)	0.0342 (0.0366)	−0.0435 (0.0235)	0.0210 (0.0366)
cggdp	−0.0007 (0.0013)	0.0025 (0.0020)	0.0008 (0.0013)	0.0012 (0.0018)
cratio2	0.0038* (0.0021)	−0.0041 (0.0033)	0.0048** (0.0023)	−0.0031 (0.0034)
cratio3	0.0012 (0.0022)	−0.0048 (0.0033)	0.0051** (0.0023)	−0.0049 (0.0035)
Individual fixed effects	Control	Control	Control	Control
Year fixed effects	Control	Control	Control	Control
Sample size	44,683	44,683	44,458	45,150
*R*-square	0.0001	0.0040	0.0000	0.0015

### 7.3 Educational disparities

The impact of residents’ educational levels on their health status is multifaceted, with educational attainment indirectly affecting lifestyle, work environment, and health behavior among Chinese residents. Due to constraints in resources and capabilities, individuals with higher levels of education tend to possess richer health knowledge, engage more easily in high-paying and low physical-demanding jobs, and enjoy better working environments and conditions, thereby experiencing lower risks of physical fatigue and illness. Conversely, residents with lower levels of education are more likely to engage in manual labor or work in unfavorable environments, making them prone to health issues.

Through indirect effects, individuals with lower educational attainment often prioritize livelihood and daily needs, leaning toward short-term behaviors and lacking sufficient environmental awareness and skills, thus overlooking the long-term impacts of their environment. Table 11 measures the changes in health status among two groups: those with a junior high school education or below and those with a high school education or above, following the implementation of EPT policies. The grouping rationale stems from the fact that in China, completing junior high school is considered the completion of basic educational requirements, as the nine-year compulsory education system in China ends with junior high school graduation.

**Table 11:** Disparities between those with junior high school education or below and those with high school education or above.

Variable	Junior high school education or below		High school education or above	
	Isdisease	Health	Isdisease	Health
	(1)	(2)	(3)	(4)
DID	−0.0257*** (0.0085)	0.0338*** (0.0123)	−0.0119 (0.0245)	0.0187 (0.0304)
Married	0.0063 (0.0151)	0.0457* (0.0241)	−0.0004 (0.0483)	0.1282** (0.0616)
Male	0.0125 (0.0586)	0.0652 (0.1083)	0.0168 (0.0839)	0.1546 (0.1170)
Age	−0.0001 (0.0008)	0.0005 (0.0014)	0.0050 (0.0044)	0.0073 (0.0090)
Educated	0.0220*** (0.0045)	0.0032 (0.0062)	0.0225 (0.0228)	−0.0303 (0.0276)
clngdp	−0.0776*** (0.0162)	0.0412 (0.0276)	−0.0046 (0.0469)	−0.0828 (0.0677)
cggdp	0.0006 (0.0010)	0.0006 (0.0014)	−0.0015 (0.0025)	0.0045 (0.0033)
cratio2	0.0037** (0.0016)	−0.0065*** (0.0025)	0.0085* (0.0043)	0.0021 (0.0063)
cratio3	0.0017 (0.0017)	−0.0074*** (0.0025)	0.0064 (0.0044)	0.0009 (0.0066)
Individual fixed effects	Control	Control	Control	Control
Year fixed effects	Control	Control	Control	Control
Sample size	77,774	78,817	10,825	11,016
*R*-square	0.0130	0.0009	0.0043	0.0039

The first two columns of Table 11 refer to middle-aged and older adult individuals with an educational level of junior high school or below, while the third and fourth columns refer to those with an educational level of high school or above. The results indicate that overall, following the implementation of EPT policies, the prevalence of chronic diseases among middle-aged and older adult individuals gradually decreased, and self-rated health levels gradually improved. The coefficients of the core explanatory variable DID in the first and second columns are significant at the 1% level, with estimated coefficients of −0.0257 and 0.0338, respectively. However, the coefficients of the explanatory variable DID in the third and fourth columns are not significant, indicating that the impact of EPT policies on improving the health status of individuals with lower educational levels is more significant.

These results suggest that EPT influence health status through environmental channels. Relative to individuals with higher educational attainment, those with lower educational levels have limited autonomy in choosing their work environment. Nevertheless, EPT can significantly improve the overall urban environment, thereby enhancing the health status of middle-aged and older adult individuals with lower educational levels.

## 8 Conclusion

This study employs the implementation of the EPT policy in 2018 as a quasi-natural experiment to assess its specific effects on the health status of middle-aged and older adult individuals. The research findings indicate that the implementation of the EPT significantly improves the self-rated health status of middle-aged and older adult individuals and reduces the incidence of chronic diseases. Moreover, after robustness checks and the elimination of other interfering factors through various methods, the results remain robust. The EPT policy enhances the health status of middle-aged and older adult individuals through two channels: the “emission reduction effect” and the “psychological effect” with the latter further divided into direct and indirect psychological effects, all of which are supported to a certain extent. The reduction in pollutants such as PM10 particles and sulfur dioxide improves regional air quality. The EPT policy is conducive to regulating residents’ emotions, improving sleep quality and memory conditions, and increasing the dissemination and attention of environmental news.

Finally, by analyzing different groups (urban–rural divide, middle-aged vs. older adult, individuals with different levels of education), the article further explores the heterogeneity of the effects of EPT policies. It is found that due to the differential impact of environmental pollution on various social groups’ health, there are differences in the mechanisms through which EPT policies affect different groups. The EPT significantly improves the chronic disease status of rural residents, the self-rated health status of urban residents, and the health status of older adult individuals and those with a junior high school education or below.

This study confirms the importance of environmental policy in promoting public health. Despite providing many valuable findings and policy recommendations, there are still some limitations and shortcomings that require further research and improvement. Firstly, the environmental protection tax policy involves multiple factors, including tax rate settings, collection methods, and the implementation strength of local governments. This paper may not have fully considered the impact of these complex factors when evaluating policy effectiveness. Further research could reveal these differences through more detailed analysis. Secondly, there are many primary indicators to measure the health levels of middle-aged and older adult. This study uses data from the China Health and Retirement Longitudinal Study (CHARLS), selecting representative indicators from the survey questionnaire. Although the data has a certain level of authority, the objectivity and authenticity of the survey questionnaire need to be further enhanced. Subsequent research can pay attention to newer and better data. Finally, the impact of the environmental protection tax on health may gradually emerge in the long term. Short-term data may not fully reflect the long-term effects of the policy. Therefore, more long-term follow-up data is needed to verify the stability and durability of the research conclusions.

This study clarifies the health effects of the environmental protection tax, its impact pathways, and population differences, having multiple policy implications. Firstly, it improves public health levels. By reducing pollutant emissions, the environmental protection tax effectively improves air and water quality, reducing the exposure of middle-aged and older adult to harmful substances, thereby lowering the incidence of chronic diseases such as respiratory and cardiovascular diseases. Secondly, it promotes ecological environmental protection. As a market-oriented environmental regulation tool, the environmental protection tax forces enterprises to internalize environmental costs. Through the “emission reduction effect” channel, it improves the health levels of the middle-aged and older adult, helping to curb the trend of environmental quality deterioration. Thirdly, it promotes social equity. The harm of environmental pollution to health often manifests more significantly in socially disadvantaged groups. Middle-aged and older adult, as physically weaker groups, are more affected by pollution. The environmental protection tax has different impacts on urban and rural areas, middle-aged and older adult populations, and people with varying educational levels, thus promoting social equity. Fourthly, it advances environmental policy innovation. The environmental protection tax features “national baseline with local flexibility,” allowing governments to formulate more flexible and precise environmental protection tax policies based on the specific circumstances of different regions, further enhancing the effectiveness and targeting of the policy. Finally, it provides international reference experience. As the largest developing country and the largest sulfur dioxide emitter in the world, China’s implementation and effectiveness of the environmental protection tax policy hold significant reference value for other developing countries.

## Data Availability

Publicly available datasets were analyzed in this study. This data can be found: https://charls.charlsdata.com/pages/data/111/zh-cn.html.
